# Equipment for eye care

**Published:** 2010-09

**Authors:** Ingrid Mason, Wanjiku Mathenge

**Affiliations:** CBM Capacity Development Officer and Medical Advisor, PO Box 58004, 00200 City Square, Ring Road Parklands, Nairobi, Kenya.; Regional Medical Advisor, Fred Hollows, Foundation. Email: clku@email.com

**Figure:**
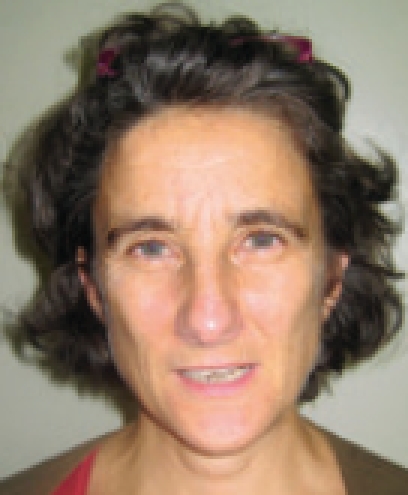


**Figure:**
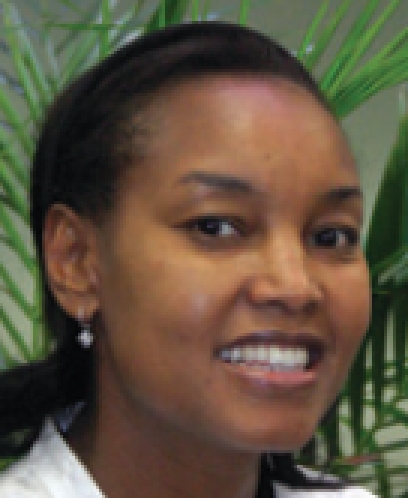


In many low- and middle-income countries, it is often the people who are poor or with a disability-or both-who find it most challenging to access and pay for health care. When people do come to us for eye care, it is therefore vital that we provide quality services efficiently and effectively.

To achieve this goal, we must ensure that our equipment is well maintained and that we have enough spare parts and consumables for it to function with minimum interruptions. To cope with the sometimes inevitable breakdowns, we also need systems that will respond quickly to carry out repairs and replace broken or worn-out parts.

Unfortunately, the survey commissioned by this journal (page 23) has shown that many eye units in low- and middle-income countries have vital equipment that is not working, often for long periods of time, and that this has affected the services offered to patients.

Equipment needs to form part of our planning for eye care. This must start when eye care programmes are being designed and should include those who will be using the equipment. Without careful planning, it is likely that our equipment will not perform optimally and might even fail completely. And without working and effective equipment, our eye care programmes will not achieve their potential.

**Figure:**
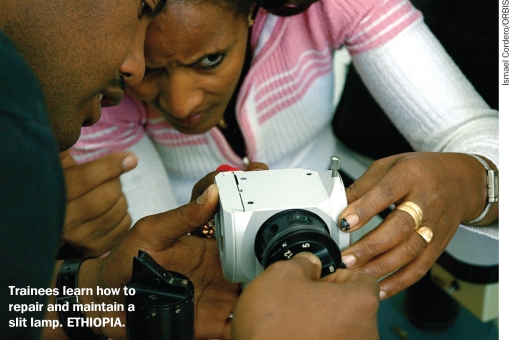
Trainees learn how to repair and maintain a slit lamp. ETHIOPIA.

Ismael Cordero/ORBIS

## Making the best out of an investment in equipment

We should critically assess whether investment in a new piece of equipment will add value to the services we offer. Does it allow the eye care team to provide a better quality service? Does it allow the team to help more patients per day? Does it help the clinician to work more comfortably (and therefore more quickly)?

The desire for sophisticated equipment should be balanced against the need for basic public health equipment such as ophthalmoscopes (as mentioned on page 32).

Ultimately, the deciding factor must be our patients. What equipment will allow the eye unit to help the largest number of patients and provide them with the best possible care?

When facing difficult decisions on a limited budget, it may be helpful to look at patient flow within the eye unit or eye care programme. Where are the longest queues, the longest delays, or the longest waiting times? These are the areas where additional investment in equipment may be of most benefit, provided everything else, including staff, is in place to support the equipment. For example, in some eye clinics there may be a queue of patients waiting to be examined at the slit lamp. One extra slit lamp may then allow the clinical staff to see many more patients per day, whereas one extra laser will not make much difference. Or in a clinic where ophthalmoscopes are shared between clinicians, a few extra ophthalmoscopes will have a similarly positive effect on patient flow.

## Budgeting and planning

The costs and expected benefits of investing in an item of equipment need to be carefully considered and put into a business plan by the eye unit manager before purchasing goes ahead. It is not always true that investing in a piece of equipment will improve productivity and outcomes! Developing a business plan will help the manager and team evaluate the costs and benefits in a rational and logical manner before taking any decisions.

Plan for installation, training (of users and the maintenance team), maintenance and repair contracts (where needed), procurement of essential spare parts and consumables, and the physical requirements of the equipment (space, temperature, and water and electricity supply).

The plan should contain the purchase cost and the cost of delivery, customs clearance, setup, and training, as well as yearly budgets for spare parts, consumables, maintenance, and repair.

## The importance of training

The sharing of technical knowledge should become part of the eye care team's normal way of working.

It is important to assign some of the responsibility for this to an equipment person or manager who will ensure that the necessary technical knowledge is shared with both users and the equipment maintenance and repair team. Training of users and the equipment team is fundamentally important to the successful use and potential impact of equipment (page 30). In turn, those who have been trained have a responsibility to pass on their knowledge to others who need it, until everyone in the eye unit has at least a basic understanding of the equipment in use.

## Relationship development with industry

Recently, some equipment manufacturers have enrolled the assistance of end-users in low- and middle-income countries to clearly outline the specifications for equipment in such environments. Another venture is the training of biomedical technicians in low- and middle-income countries in the installation, care and maintenance of their equipment.

This positive partnership between VISION 2020/IAPB consortium partners, eye care programmes, and end-users demonstrates how careful and creative thinking can benefit both end-users and equipment manufacturers.

In conclusion, if we are to meet the goals of VISION 2020, we as eye care providers must acknowledge the potential of equipment to contribute to these goals -and plan accordingly.

